# Performance of Winter-Sown Cereal Catch Crops after Simulated Forage Crop Grazing in Southland, New Zealand

**DOI:** 10.3390/plants10010108

**Published:** 2021-01-06

**Authors:** Brendon Malcolm, Shane Maley, Edmar Teixeira, Paul Johnstone, John de Ruiter, Hamish Brown, Stewart Armstrong, Steven Dellow, Mike George

**Affiliations:** 1 The New Zealand Institute for Plant and Food Research Limited, Christchurch 7647, New Zealand; shane.maley@plantandfood.co.nz (S.M.); edmar.teixeira@plantandfood.co.nz (E.T.); john.deruiter@plantandfood.co.nz (J.d.R.); hamish.brown@plantandfood.co.nz (H.B.); steven.dellow@plantandfood.co.nz (S.D.); mike.george@plantandfood.co.nz (M.G.); 2 The New Zealand Institute for Plant and Food Research Limited, Hastings, Havelock North 4172, New Zealand; paul.johnstone@plantandfood.co.nz; 3 The New Zealand Institute for Plant and Food Research Limited, Gore 9774, New Zealand; stewart.armstrong@plantandfood.co.nz

**Keywords:** *Avena sativa* L., *Secale cereale* L., *Triticosecale*, nitrate leaching, kale, fodder beet

## Abstract

(1) Background: Winter grazing of livestock poses significant environmental risks of nitrogen (N) leaching and sediment runoff. (2) Methods: A field study tested the effects of sowing catch crops of oats (*Avena sativa* L.), ryecorn (*Secale cereale* L.) or triticale (*Triticosecale*) in June and August (winter) in Southland, New Zealand (NZ), on the risk of N leaching losses from simulated N loads left after winter forage grazing. (3) Results: Catch crops took up 141–191 kg N ha^−1^ by green-chop silage maturity (approximately Zadoks growth stage 52; November/December). Importantly, early-sown catch crops were able to capture more N during the key leaching period from winter to mid-spring (77–106 kg N ha^−1^ cf. 27–31 kg N ha^−1^ for June and August treatments, respectively). At this time, ryecorn and triticale crops sown in June captured 20–29 kg ha^−1^ more N than June-sown oats (77 kg N ha^−1^). In October, early-sown catch crops reduced mineral N in the soil profile (0–45 cm depth) by 69–141 kg N ha^−1^ through the process of plant uptake. At green-chop silage maturity, catch crop yields ranged from 6.6 to 14.6 t DM ha^−1^. Highest yields and crop quality profiles (e.g., metabolizable energy, crude protein, soluble sugars and starch) were achieved by the oats, irrespective of the sowing date, indicating that trade-offs likely exist between environmental and productive performances of the catch crop species tested. (4) Conclusion: The catch crop of choice by farmers will depend on the desired end use for the crop, its place in the crop rotation and its potential for an environmental benefit.

## 1. Introduction

Forage kale (*Brassica oleracea* var. *acephala* L.), swedes (*Brassica napus* ssp. *napobrassica*) and fodder beet (*Beta vulgaris* L. ssp. *vulgaris*) are important single-graze species used for feeding livestock during winter months when pasture growth rates are typically low, particularly in cooler climatic regions of New Zealand (NZ), such as Canterbury, Otago and Southland [1]. In these systems, land often remains fallow for periods of up to five months post-grazing, until a new crop is established in spring [2]. Livestock urine is the major source of nitrogen (N) leaching in these systems [3], and because urine deposition coincides with periods of high rainfall and greatest rates of drainage (winter) when there is absence of an active plant sink to take up residual N, this represents a significant environmental risk in terms of N loss [4]. Nitrate-N (NO_3_^−^-N) leaching losses of between 30 and 180 kg N ha^−1^ have been measured from winter grazing systems [5,6,7,8] and are largely influenced by seasonal factors, such as the timing and amount of rainfall [9]. 

Catch crops (short-term crops established between two main crops) have recently been investigated in NZ for their potential to “mop up” residual soil mineral N (SMN) and reduce the risk of N leaching losses from these grazing systems [10,11,12,13,14]. Carey et al. [10] used monolith lysimeters to study effects of winter grazing dates and catch crop sowing timings and reported reductions in NO_3_^−^-N leaching losses averaging 34% under oat (*Avena sativa* L.) catch crops compared with fallow conditions. Similarly, through field plot experimentation, Malcolm et al. [13] reported up to 243 kg N ha^−1^ captured in above-ground biomass by oats (harvested in November) when sown in July or August, which resulted in significant reductions in SMN in spring. 

Catch crop selection and timing of growth are important considerations for effective N uptake before leaching out of the root zone, given the short window of opportunity for establishment following winter grazing. For instance, winter catch crops must germinate and establish under often unfavorable soil and climatic conditions, develop an extensive root system, and actively grow and take up N under particularly cool temperatures. Cereals, namely, oats and triticale (*Triticosecale*), have largely been preferred because of their relatively high winter activity and inherently large sink for N [14,15,16,17]. For instance, Carey et al. [15] showed that winter-sown oats reduced NO_3_^−^-N leaching by 25% compared with Italian ryegrass (*Lolium multiflorum* Lam.). On a global scale, a meta-analysis by Thapa et al. [18] highlighted that non-leguminous species sown late summer–autumn were more effective cover crops than leguminous monocultures for reducing NO_3_^−^-N leaching, likely to also be the case with winter-sown catch crops. Ryecorn (*Secale cereale* L.) is a crop that can establish well under cool temperatures, and in some earlier work, ryecorn was shown to exhibit greater cool season growth than oats. In the study of McDondald and Stephen [19] in the Otago region of NZ, ryecorn sown in autumn yielded 1.8, 3.3 and 6.3 t DM ha^−1^ in August, September and October, respectively, compared with 0.6, 1.5 and 4.6 t DM ha^−1^ by oats. However, the authors also noted that during a subsequent wetter season, ryecorn yields were suppressed and at times outperformed by oats.

In cooler regions of NZ such as Southland, cool temperatures are likely to result in variable catch crop performance following winter sowings. Oats typically perform well under cool conditions; however, other winter-active cereals may confer advantages for N accumulation in the herbage. The objective of this study was to test the effectiveness of oats, ryecorn and triticale, sown in winter (June or August), on reducing the risk of N leaching losses from winter-grazed forage crops in Southland. 

## 2. Results and Discussion

### 2.1. Climate and Drainage

**Figure 1:**
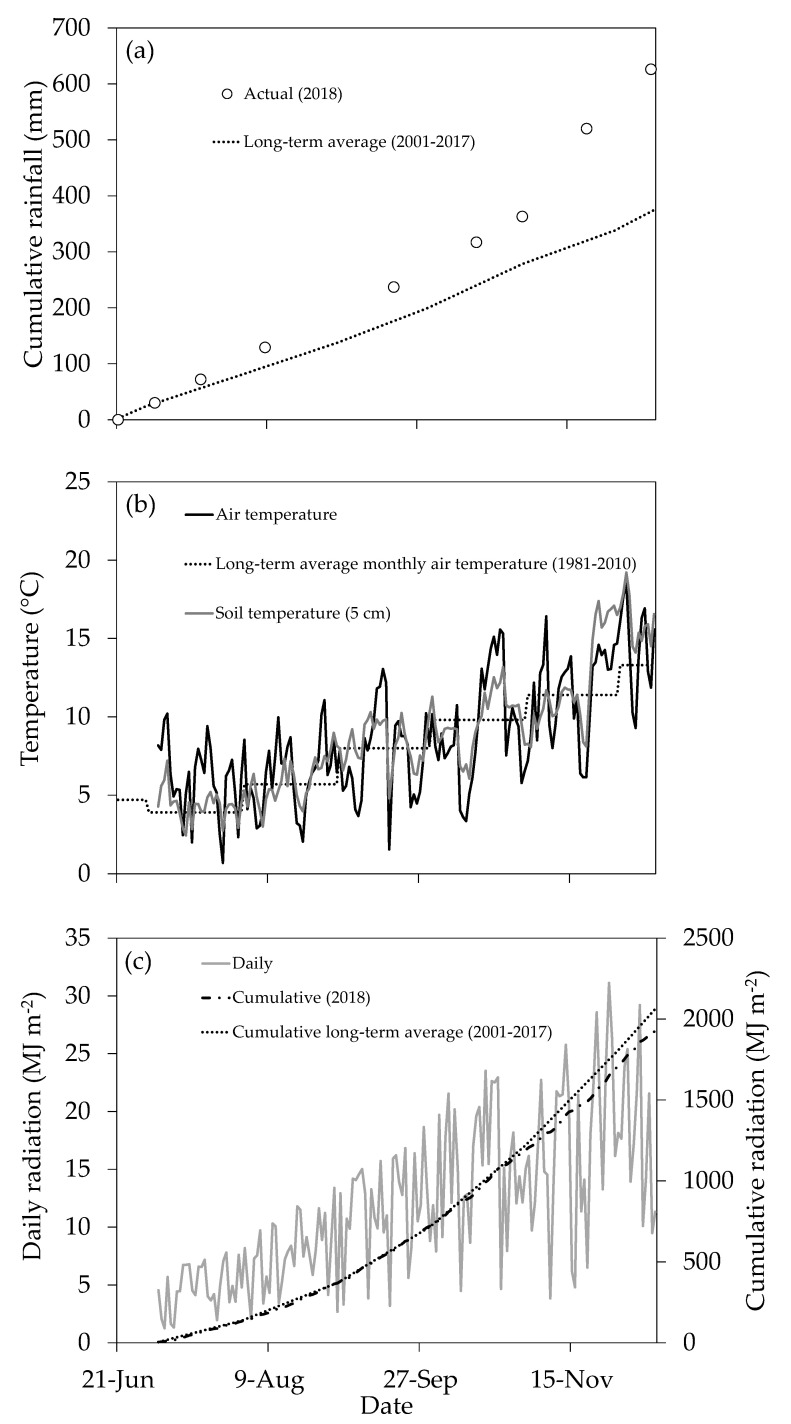
Rainfall (**a**), air temperature and soil temperature (**b**) and solar radiation (**c**) patterns collated for the trial period (22 June to 12 December 2018) at the Mossburn trial site, Southland.

The 2018 winter–spring period at the site was substantially wetter than normal. The total amount of rainfall received during the trial period (June–December) was 626 mm, approximately 250 mm more than the long-term average (Figure 1a). In general, daily mean air temperatures were similar to long-term averages; however, in July the daily means averaged 6 °C, about 2 °C warmer than the long-term average (Figure 1b). Cumulative daily solar radiation reached almost 2000 MJ m^−2^ during the trial period, with daily values generally consistent with the long-term average between the beginning of July and the end of October. Daily solar radiation was 2.5 MJ m^−2^ per day lower than average during November and early December, coinciding with the particularly wetter than average months (Figure 1c). Such weather conditions might imply trade-offs for risk of leaching in relation to historical means. For instance, warmer and wetter conditions can increase the risk of leaching due to more frequent drainage events and also faster soil N mineralization rates. However, the same conditions accelerate catch crop growth, which might result in greater N uptake, reducing the risk of leaching. 

**Figure 2:**
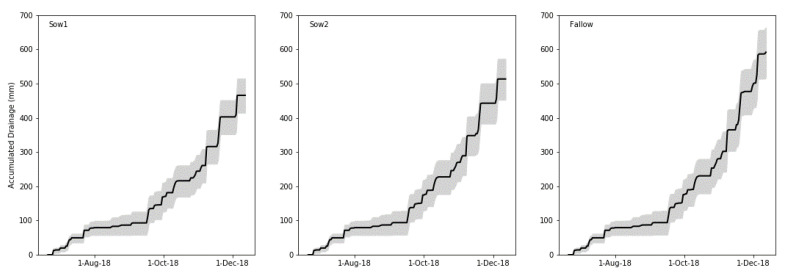
Estimated accumulated drainage under catch crops sown on 21 June 2018 (Sow1) and 8 August 2018 (Sow2) and fallow soil at Mossburn, Southland, New Zealand. The gray shading represents a range of drainages estimated assuming differing parameters in the soil water balance equations.

Figure 2 shows the estimated cumulative drainage over the trial period. A total of approximately 600 mm of drainage was recorded under the fallow control by December, 20–25% more than that estimated from the June and August catch crop sowing date treatments. Lower drainage estimated from catch crops was attributed to greater rates of evapotranspiration, which was an important mechanism of reduced N leaching losses in previous studies [10,18].

### 2.2. Crop Development, Nitrogen and Feed Quality

**Figure 3:**
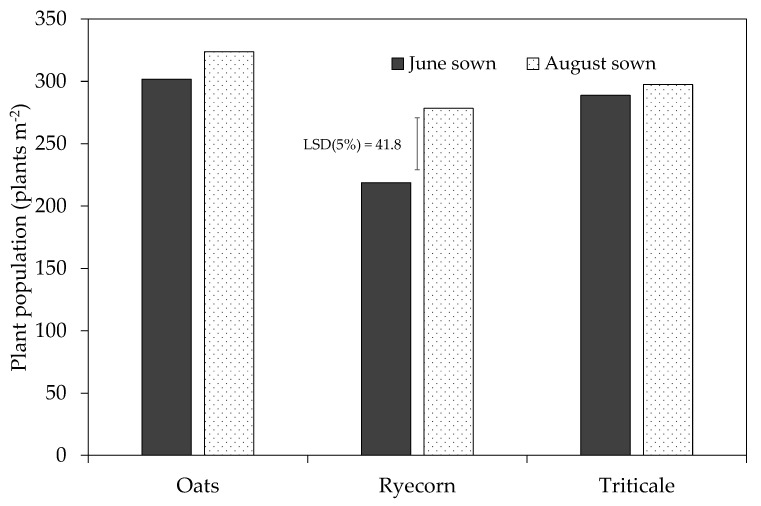
Plant population (plants m^−2^) of catch crop treatments at the Mossburn trial, Southland, in 2018. A single application of urea fertilizer (400 kg N ha^−1^) was applied to all plots on 21 June (simulating N loading within a cow urine patch). The thin vertical bar represents the least significant difference (LSD) at the 5% level, *n* = 4.

With the exception of June-sown ryecorn (219 plants m^−2^), all treatments emerged and were near target populations of 300 plants m^−2^ (278–324 plants m^−2^) at 48 and 42 days after sowing (DAS) for June- and August-sown treatments, respectively (Figure 3). The slow rate of emergence could be expected, given the cool winter temperatures (Figure 1b) and a thermal time requirement of 132 degree-days for “Milton” oats to reach 75% emergence [16]. There was a highly significant (*P* = 0.001) and significant (*P* = 0.018) main treatment effect of catch crop species and sowing date on plant population, respectively. For instance, populations were on average 21% and 15% less in ryecorn treatments compared with oats and triticale, respectively, and 27% more for ryecorn when sowings were delayed until August compared with those sown in June. Overall, these populations are in line with those reported in a field trial in Canterbury by Malcolm et al. [20], where populations of up to 276 plants m^−2^ were recorded. 

**Figure 4:**
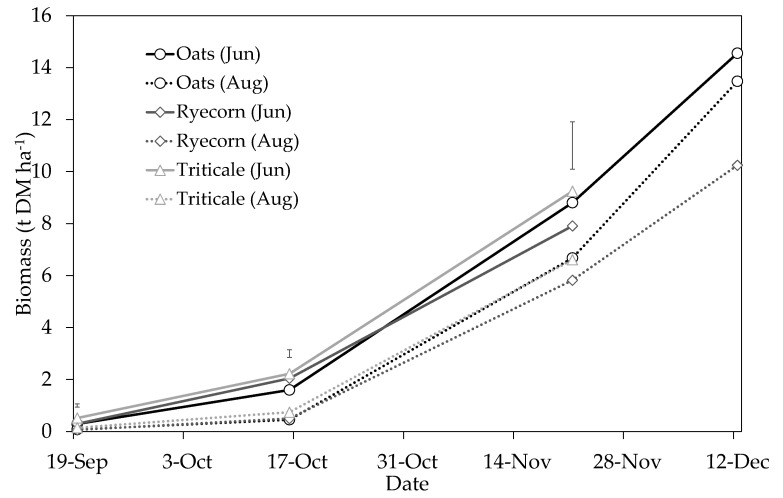
Cumulative above-ground biomass (t DM ha^−1^) of catch crops sown on 21 June (Jun) or 8 August (Aug). The sampling on 21 November represents approximate green-chop silage maturity for “Ryecorn (Jun)”, “Triticale (Jun)” and “Triticale (Jul)”, while samples obtained on 12 December represent approximate green-chop silage maturity for all other treatments. A single application of urea fertilizer (400 kg N ha^−1^) was applied to all plots on 21 June (simulating N loading within a cow urine patch). Vertical bars represent the least significant difference (LSD) at the 5% level for the initial three samplings, *n* = 4.

There were highly significant (*P* < 0.001) main treatment effects of sowing date on DM production at each of the initial three harvests (Figure 4). Similarly, there were significant main treatment effects of catch crop species on DM production at the 19 September 2018 (*P* = 0.003) and 16 October 2018 (*P* < 0.001) samplings. Overall, DM yields at each of the initial three harvests were 1.4–3.9-fold greater in the June-sown treatments than the respective August-sown treatments. This was largely due to the longer growing period that enabled crops to intercept more photosynthetically active radiation (PAR) for biomass accumulation. This effect of sowing date was clearly demonstrated in the studies of Martini et al. [21] and Zyskowski et al. [22], where progressively lower growth rates and DM yields were recorded with every month that sowing of oats (between March and June) was delayed. Similarly, in a field experiment in Canterbury, Malcolm et al. [13] showed the benefits of sowing catch crops early and reported winter-sown oat DM yields similar to ours under simulated urine patch conditions (0.5–1.0 and 2.0–5.4 t DM ha^−1^ in October and November, respectively, during a largely cooler than average Canterbury season).

During the earlier stages of growth (initial two harvests), there was evidence that June-sown triticale and ryecorn crops accumulated more biomass than oats. For example, at the 19 September sampling, triticale had accumulated on average 73 and 77% more DM than oats and ryecorn (*P* < 0.05; equating to an additional 0.23 and 0.22 t DM ha^−1^, respectively). At the second harvest (October), triticale and ryecorn yielded on average 44% and 24% more DM than oats (1.6 t DM ha^−1^), respectively (*P* < 0.05). This was attributed to the likely lower thermal time requirements of vegetative development for ryecorn and triticale [23] and, consequently, a higher level of early vigor. However, at a later stage (green-chop silage maturity), oats sown in June and August had accumulated >13 t DM ha^−1^, compared with ≤10.2 t DM ha^−1^ for the other species. We attributed the lower triticale and ryecorn yields at green-chop (approximately Zadoks growth stage 52) to the earlier onset of inflorescence (shorter vegetative phase) of both these species, i.e., the June- and August-sown triticale and June-sown ryecorn crops were harvested at green-chop silage maturity 21 days earlier than oats. This highlights the importance of timing in catch crop growth depending on the agronomic targets (e.g., timing of sowing of next main crop) of the production system in study.

**Figure 5:**
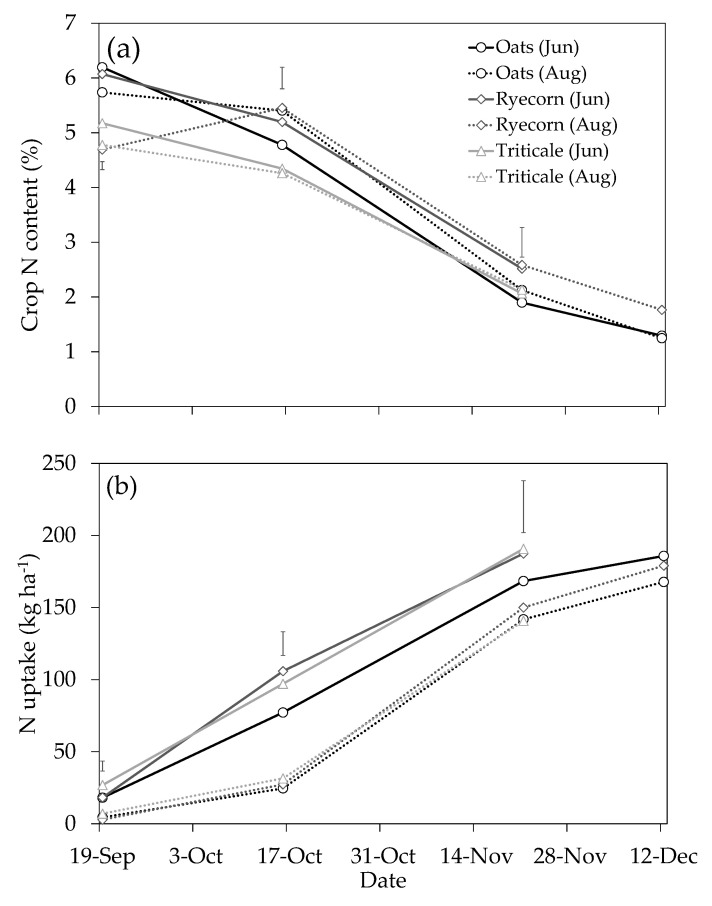
Crop nitrogen (N) content (%) (**a**), and cumulative crop N uptake (kg ha^−1^) (**b**) for catch crops sown on 21 June (Jun) or 8 August (Aug) at the Mossburn trial, Southland, in 2018. The sampling on 21 November represents approximate green-chop silage maturity for “Ryecorn (Jun)”, “Triticale (Jun)” and “Triticale (Jul)”, while samples obtained on 12 December represent approximate green-chop silage maturity for all other treatments. A single application of urea fertilizer (400 kg N ha^−1^) was applied to all plots on 21 June (simulating N loading within a cow urine patch). Vertical bars represent the least significant difference (LSD) at the 5% level for the initial three samplings, *n* = 4.

At the initial two samplings in September and October, crop N contents ranged between 4.2% and 6.3% for all treatments (Figure 5a), with significant (*P* < 0.001, September; *P* < 0.05; October) interactions observed between crop species and sowing date. Nitrogen content was notably less at subsequent samplings, due to a dilution effect during periods of higher growth rates. Earlier work by Eagles et al. [24] in Manawatu also showed a steady decline in N concentration over the course of the growth period of various oat cultivars (4.5% N in June, declining to 1% in October). June-sown ryecorn and triticale saw 26–37% more N captured by October than that in June-sown oats (Figure 5b), corresponding to the greater amount of biomass accumulation during early stages of development. By 21 November, between 141 and 191 kg N ha^−1^ was captured by all catch crops species, reducing the amount of N from the leachable pool. There was a strong and highly significant (*P* ≤ 0.001) effect of sowing date on N uptake at each of the initial three samplings, with higher rates of N uptake in the earlier-sown crop treatments. This effect was particularly evident at the 16 October sampling, which in general represents when drainage/leaching is likely to be declining.

The suite of crop quality components measured at green-chop silage maturity is given in Table 1. There were significant interactions between sowing date and species treatments for all crop quality components, except crude product (CP), soluble sugars and starch (SSS) and hemi-cellulose (Table 1). For these analyses, there were, however, significant main treatment effects of sowing date and species, suggesting crop choice and agronomic management practices will likely have significant economic outcomes in terms of feed value. Metabolizable energy across all the treatments ranged from 8.55 to 11.53 MJ kg^−1^ DM, which is within ranges reported by de Ruiter et al. [25] for autumn- and spring-sown cereals. For each sowing date, oats had significantly (*P* < 0.05) higher (by 9–25%) ME contents than other crop species. In addition, oats were significantly (*P* < 0.001) higher than the other species in terms of SSS and crude fat, but consistently lower in crude protein and fiber (neutral detergent fiber (NDF) and acid detergent fiber (ADF)). The higher relative nutritive value (component indicators and metabolizable energy (ME)) of oats is an important consideration for farmers as this crop was also higher yielding at the green-chop stage and therefore produced substantially greater amounts of harvested ME for both sowing dates (Table 1). The potential environmental gains from June-sown oats, however, are evidently less than from ryecorn or triticale because of the lower protein content on the basis of equivalent yield (also refer to Figure 5). The timing of N uptake in relation to rainfall events and N leaching pattern also needs to be considered relative to potential nutritive value gains with species choice.

### 2.3. Soil Mineral Nitrogen

**Figure 6:**
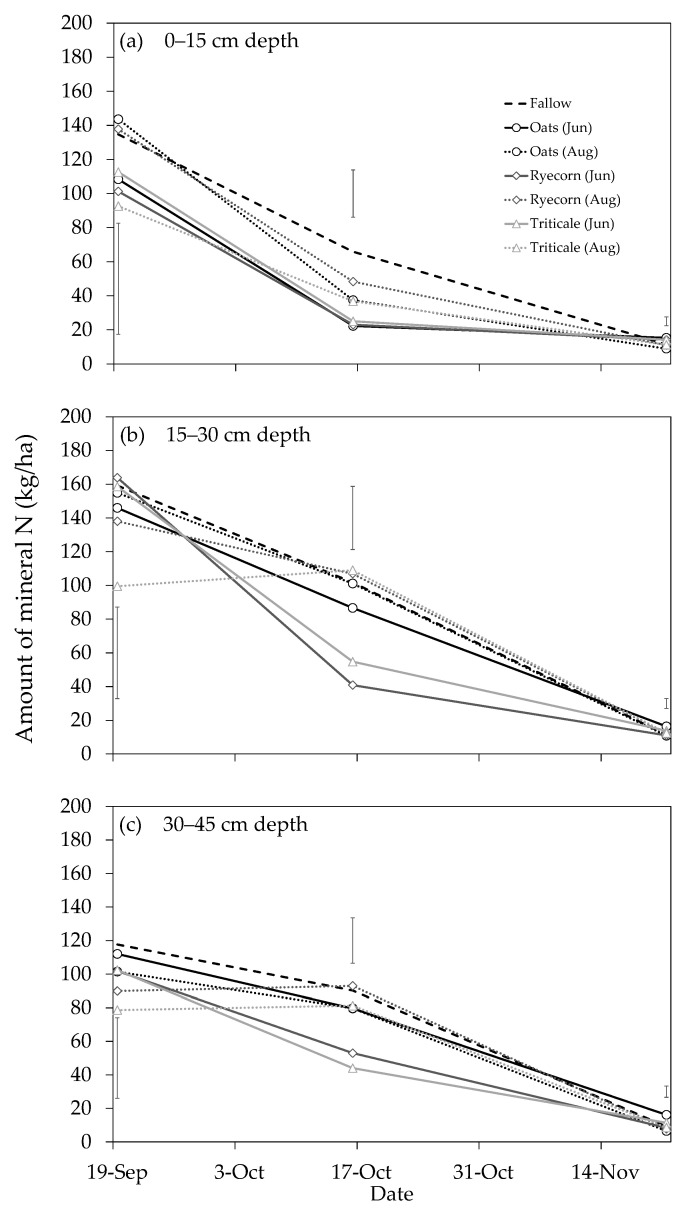
Soil mineral nitrogen (N; kg ha^−1^) at 0–15 (**a**), 15–30 (**b**) and 30–45 cm depth (**c**) for catch crops sown on 21 June (Jun) or 8 August (Aug) and a fallow control treatment, for the Mossburn trial, Southland, in 2018. A single application of urea fertilizer (400 kg N ha^−1^) was applied to all plots on 21 June (simulating N loading within a cow urine patch). Vertical bars represent the least significant differences (LSD) at the 5% level, *n* = 4.

Initial SMN concentrations prior to catch crop sowing (21 June 2018) were between 15 and 30 kg N ha^−1^ (data not shown). The 400 kg N ha^−1^ applied on 21 June (to simulate urine-N loading during forage crop grazing) would have represented a substantial increase in the amount of N in the soil profile. This is evident from the notably larger SMN concentrations in the soil at the 19 September sampling (Figure 6). In addition, increased SMN concentrations at deeper layers of the soil were observed, indicating downward movement of N, i.e., leaching, which can be expected given the amount of rainfall received leading up to the September sampling and the large volumes of estimated drainage (Figure 2). At both the 19 September and 16 October samplings, >93% of the N was NO_3_^−^-N (mobile form of N), when averaged across all treatments (data not shown). 

Although not statistically significant, there was evidence that catch crops began to reduce SMN at the 19 September sampling (Figure 6). Marginally lower amounts of SMN were measured under catch crops compared with the fallow control, consistent with findings of a similar study in Canterbury by Malcolm et al. [13]. At the 16 October sampling, there was a significant (*P* = 0.012) main treatment effect of “catch crop” on SMN (0–45 cm depth) and a highly significant (*P* < 0.001) main treatment effect of “sowing date”. There was also evidence of a reduction in SMN occurring at lower depths in the profile under all catch crops, particularly in the June-sown ryecorn and triticale treatments (41% and 51% lower than the fallow control in the 30–45 cm depth horizon, respectively; *P* < 0.05 (Figure 6c)). By 21 November, SMN in the fallow treatment reached levels similar to those at the beginning of the experiment, indicating that a significant amount of N had been lost from the system. Inevitably, some N was lost via various gaseous loss pathways, and some N immobilized via microbial activity, but much was likely to have been lost below 45 cm in drainage water, given the particularly wet season (Figure 1a) and the free-draining nature of the soil. Therefore, considering residual SMN levels in the catch crop treatments were also similar to the fallow, and given catch crops had retained between 141 and 191 kg N ha^−1^ in above-ground biomass, it is likely that the catch crops had significantly reduced N leaching losses.

### 2.4. Net Nitrogen Supply

**Figure 7:**
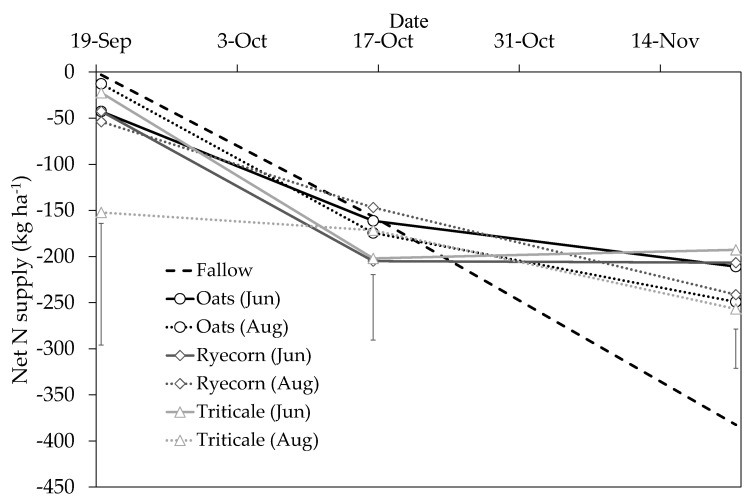
Net nitrogen (N) supply (kg ha^−1^) within the 0–45 cm depth horizon for catch crops sown on 21 June 2018 (Jun) or 8 August 2018 (Aug) and a fallow control treatment in the Mossburn trial, Southland. A single application of urea fertilizer (400 kg N ha^−1^) was applied to all plots on 21 June (simulating N loading within a cow urine patch). Vertical bars represent the least significant differences (LSD) at the 5% level, *n* = 4.

There was a net loss of N from the plant–soil system from the initial post-treatment sampling on 19 September until the final soil sampling on 21 November (Figure 7). This was attributed to the particularly wet weather conditions and the large volumes of estimated drainage, resulting most likely in N being lost through leaching, as described above. At the final soil sampling (21 November), there were highly significant effects (*P* ≤ 0.001) of “catch crop” and “sowing date” on net N supply. For example, net N supply from the June-sown catch crop treatments was 175–189 kg ha^−1^ greater (*P* < 0.05) than the fallow control (-382 kg N ha^−1^). When catch crop sowings were delayed until August, net N supply remained 126–141 kg N ha^−1^ greater than the fallow control. In both cases, these differences can be attributed to the amounts of N that were retained by the catch crops. Interestingly, at the 16 October sampling, there was a notable trend for a greater net loss of N under the June-sown catch crop treatments than other August-sown treatments and the fallow control, despite the greater amounts of N that had been taken up by the respective crops. It is unlikely that a greater amount of N leaching occurred beneath the early June-sown catch crops (because of earlier N uptake and water use by the developing crop), and therefore this greater loss is either due to enhanced gaseous losses of N (although unlikely given catch crops draw moisture and create conditions that are less conducive to denitrification), some N retention in the root systems [12] or that the catch crops were sequestering carbon to the soil via their root systems (root exudation/turnover), which in turn enhanced microbial immobilization of N. Nitrogen immobilization has been observed in previous catch crop trials [12,18] and warrants further investigation.

### 2.5. Practical Considerations

Our results imply that for the three catch crop species tested, there are possible trade-offs between their environmental performance and production potential. For example, winter-sown ryecorn and triticale are likely able to offer marginally greater environmental benefits than oats during the leaching period (winter to mid-spring) due to quick establishment and canopy development. On the other hand, oats can offer a greater amount of biomass and a better feed quality profile at green-chop silage maturity, primarily due to a longer period of vegetative growth. The selection of catch crop to grow depends on the amount of N leaching reduction required (regulatory drivers), the time available until the subsequent spring crop needs to be sown and the desired final use for the catch crop. In addition, work that considers the practical challenges of establishing catch crops after winter forage grazing in Southland is, and will continue to be, important. Recent work by Carey et al. [26] on commercially run Southland properties (following grazed kale or fodder beet) demonstrated the potential for a single-pass spader and drill combination implement. This enabled sowing of catch crops significantly earlier than conventional practices, with notable benefits in terms of DM production and N capture. Importantly, there is a significant risk of soil physical damage on some soil types when heavy machinery is used to cultivate and sow catch crops during the wet winter months, which should also be considered in future work. 

Finally, given the establishment of a ground cover at this time of the year, there might be positive spin-off effects on reducing sediment losses from these systems. Monaghan et al. [27] reported that 92% of sediment loss in hill country winter grazing systems occurs after grazing. This is also an area for further research.

## 3. Materials and Methods 

### 3.1. Site Information

The study area was a commercial dairy wintering farm in Mossburn, Southland (45°41.22′ S, 168°15.90′ E). The soil was a shallow Morven silt loam (soil order “Brown”) [28], with a plant available water-holding capacity of approximately 60 mm m^−1^ of depth. Prior to the experiment, the trial area had been under a cereal wheat grain crop that was harvested in February 2018. Background soil fertility (0–15 cm depth) of the general trial area is given in Table 2. All nutrients were within optimum range, except that sodium (Na) was less. The low Na was unlikely to have influenced the results, given soil Na is generally not yield-limiting and is often substituted by potassium [29]. In preparation for the trial, on 1 April 2018, the general trial area was ploughed, power-harrowed and excluded from grazing livestock.

### 3.2. Trial Design and Treatments

The experiment was a row-column design, with three catch crop species treatments (“Milton” oats, “Rahu” ryecorn and “WinterMax” triticale), two sowing dates (June and August 2018) and a fallow control. Each treatment consisted of four replicate plots. Cultivars were selected with assistance from local seed suppliers who were familiar with the conditions in the region and had knowledge of the likely best winter-performing options.

Individual plot dimensions were 10 m long × 1.95 m wide (nine 13 cm rows). The early sowing treatments were sown with a 1.95 m-wide Taege direct drill at a target population of 300 plants m^−2^ on 21 June 2018 (oats 111 kg seed ha^−1^; ryecorn 126 kg seed ha^−1^ and triticale 165 kg seed ha^−1^), and the late sowing was on 8 August 2018 at the same seeding rates. 

On the same day that the first catch crop treatments were sown (21 June), a single rate of 400 kg N ha^−1^ as SustaiN^®^ urea fertilizer (46% N) was evenly applied to all plots, i.e., to both sowing date treatments and the fallow control. The high N rate of 400 kg N ha^−1^ was chosen to simulate N loading from dairy cow urine patches deposited during winter grazing of the preceding crop [13,20]. The N fertilizer was broadcast using a manually operated hand spreader and lightly incorporated using harrows.

All crops were managed as per current industry practice. This included the use of selective herbicides to control volunteer weeds in catch crop plots and non-selective herbicides to maintain a weed-free status of the fallow plots. Aside from the N applied to simulate urine patches, no other fertilizer was applied for the duration of the experiment.

### 3.3. Measurements

The number of emerged seedlings within two side-by-side rows of a randomly placed 1 m strip was counted in each of the June-sown plots. Counts were performed on 8 August and 10 September for the August-sown plots, 42–48 DAS. This information was used to calculate plant populations.

Crop biomass and N uptake patterns were measured on 19 September, 16 October and 21 November 2018 for all crop treatments. Additional harvests were performed on 12 December 2018 for both June- and August-sown oats and August-sown ryecorn to align with green-chop silage maturity (approximately 50% panicle/ear emergence, i.e., Zadoks growth stage 52). On each sampling occasion, above-ground biomass was measured in each plot from a single 0.5-m^2^ quadrat. Cuts were taken at ground level and then weighed fresh. A subsample (approximately 400 g) was oven dried at 60 °C for approximately 48 hours (or until a constant dry weight was achieved) to determine percent dry matter (DM), then ground and analyzed for total N content using a LECO CNS analyzer (LECO Corporation, St Joseph, MI, USA). Dry matter yield and N concentration in the tissue were used to determine crop N uptake on each sampling occasion. Nutritive value components of additional freeze-dried and finely ground crop samples, taken at the green-chop silage maturity stage, were determined using recognized in vitro methodologies (Hill Laboratories) and consisted of metabolizable energy (ME), crude protein (CP), acid detergent fiber (ADF), neutral detergent fiber (NDF), lignin, soluble sugars and starch (SSS), digestibility (disappearance of organic matter in dry matter; DOMD), crude fat and hemi-cellulose. No biomass measurements were taken from the fallow plots.

Soil mineral N (sum of ammonium-N (NH_4_^+^-N) and NO_3_^−^-N) was measured across the general trial area at the start of the experiment on 21 June 2018 (before N fertilizer application). Each plot was then further measured on 19 September, 16 October and 21 November. Each sample consisted of composite soil cores collected from 0–15, 15–30 and 30–45 cm depths, using a hand-operated Dutch auger. Sampling beyond 45 cm in this stony soil was too physically challenging, but we acknowledge cereal roots can extract SMN from below 45 cm depth. At each sampling, two soil samples from the same depth horizon within each plot were combined and passed through a 2-mm sieve. A well-mixed subsample of 5 g of sieved soil was taken and extracted with 2 M KCl at a 1:5 soil-to-solution ratio. The filtered extract was analyzed for NO_3_^−^-N and NH_4_^+^-N content on a Lachat QuikChem 8500 Series 2 Flow Injection Analysis System (Lachat Instruments, Loveland, Colorado, USE; Keeney and Nelson, 1982).

To determine the change in available N within the soil–plant system of each treatment over time, compared with the amount of initial available N (soil and applied N), we calculated the “net N supply” for three sampling dates (19 September, 16 October and 21 November). Net N supply (kg ha^−1^) was calculated using the following equation:

*Net N supply* = (*R* + *U*) − (*I* + *A*), (1)

where *R* represents the amount of residual SMN (kg ha^−1^), *U* is the amount (kg ha^−1^) of N in the crop (above-ground only), *I* is the initial amount of residual SMN (kg ha^−1^) before treatments were imposed and *A* is the amount (kg ha^−1^) of N added (i.e., fertilizer/simulated urine N). A positive net N supply indicates a net addition of N to the system (e.g., through mineralization and/or N fixation). A negative value indicates a net loss of N (e.g., through leaching below 45 cm depth, gaseous loss and/or immobilization). Net N supply can be used as a proxy for assessing how effective a management practice might be in retaining N within the system, and thus preventing N from being lost.

A weather station was installed at the site and air temperature, soil temperature, rainfall and solar radiation were monitored from 4 July 2018 through until the trial ceased. A manual rain gauge was also installed at the site. There was a malfunction of the rainfall sensor during the initial few weeks of the trial; therefore, we report data from the manual rain gauge data only. For long-term average values of rainfall (2001–2017) and air temperature (1982–2010), weather data were collected from the nearest NIWA weather station (Lumsden AWS; 45°44.88′ S, 168°26.94′ E) located approximately 16 km from the trial site. For long-term average solar radiation (2001–2017), data from the Gore AWS NIWA weather station (46°6.90′ S, 168°53.22′ E) were used.

Given N leaching is driven largely by drainage volume, and in the current study this was not directly measured, Python was used as a modeling platform to calculate a theoretical water balance to indicate how much drainage was likely to have occurred during the catch crop phase [11]. Daily soil water content (SWC_d_) was calculated as

*SWC_d_* = *SWC_d_*_−1_ + *R* − *T* − *E* − *D*, (2)

where *R* is rainfall, *T* is transpiration, *E* is evaporation and *D* is drainage. Drainage was calculated as

*D* = *max*{0, (*SWC_d_*_−1_ + *R* − *T* − E) − *DUL*}, (3)

where *DUL* is the drained upper limit of the profile. *T* was calculated as PET × Cover. *E* was calculated as PET × (1-Cover) × FS. PET is Priestley Taylor potential evapotranspiration calculated with an alpha coefficient of 1.3. F_s_ is a factor that accounts for bare soil having a lower evaporation than crop canopies. Cover was assumed to follow a sigmoidal pattern following sowing:

(4) $$Cover\;=\;\frac{1}{1+e^{-\left(Tt-X_{o}\right)/b}},$$

where *Tt* is the daily mean temperature accumulation from sowing, and *X_o_* and *b* were assumed to have values of 150 and 800, respectively, for all crops.

**Figure 8:**
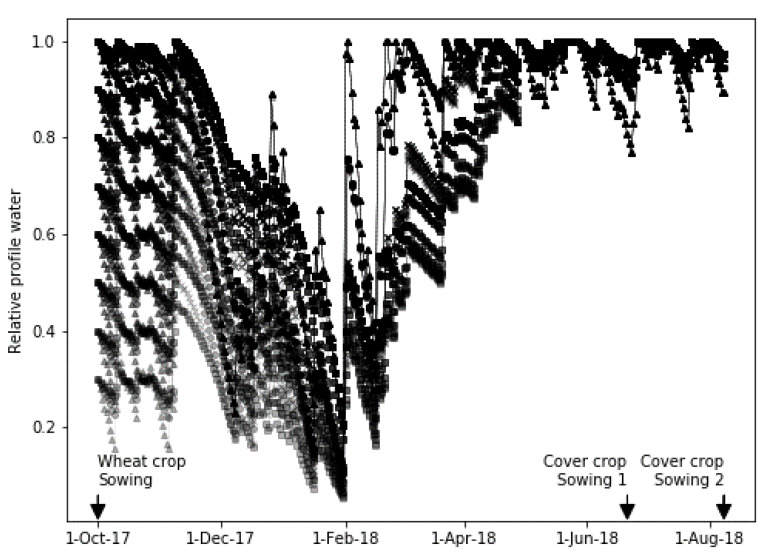
Soil water content relative to drained upper limit for a range of calculations assuming different drained upper limits and initial soil water contents.

Initial soil water content (SWC_d−1_ on the day of wheat sowing) and DUL were not known; therefore, a series of SWCd calculations were run to see what impact variation in these parameters would have on SWC_d_ on the day the catch crops were sown. A range of DUL (50–200 mm) and initial SWC (30–100% of DUL) were used and Figure 8 shows that, regardless of the value assumed for these, the SWC_d_ on the day of catch crop sowing was close to DUL (value of 1 = DUL). Thus, an SWC_d−1_ = 0.95 * DUL could be safely assumed for estimating drainage under the catch crops for both sowing dates.

Finally, a set of SWCd calculations was run over the duration of the catch crops with a range of DUL (50–200 mm) and F_s_ (0.2–1.0) parameters. 

### 3.4. Statistical Analysis

Differences in means of plant population, DM yield, N content, N uptake, SMN, net N supply and each of the feed quality components were assessed by an analysis of variance (ANOVA) using GenStat v. 17 (VSN International, Hemel Hampstead, UK). Significant interactions and main effects were separated using Fisher’s protected least significant difference (LSD) tests (α = 0.05). 

## 4. Conclusions

The main conclusions drawn from this experiment are as follows:

- In the cold climate of Southland, New Zealand, winter-sown catch crops of oats, ryecorn or triticale were shown to have potential to establish and remove residual SMN and water from the soil, reducing the risk of N leaching losses after simulated winter forage grazing. One of the challenges ahead for this practice in a working farm system is the ability to sow the catch crops into suitable seedbeds, considering the typically wet and/or pugged conditions that are often associated with winter grazing, particularly in Southland.
- Final green-chop silage maturity yields ranged from 6.6 to 14.6 t DM ha^−1^, presenting significant productivity opportunities for the use of catch crops in this context. Higher yields were achieved when crops were sown earlier in winter.
- Early sowing in winter was particularly important for capturing N during the high-risk drainage/leaching period, i.e., winter months until approximately mid-spring. Farmers should, therefore, aim to sow cereal catch crops as early as possible following winter forage crop grazing, in order to maximize both the environmental and productive benefits of catch crops.
- When sown in June, oats took up less N than ryecorn and triticale during early growth stages; however, delayed sowings until August resulted in non-significant differences between species.
- High N losses evidently occurred from this simulated winter grazing scenario. Between 19 September and 21 November, there were substantial losses of N within the soil profile (0–45 cm depth) across all treatments. This is unsurprising given the shallow, free-draining nature of the soil, the wet conditions and the large volumes of estimated drainage. The amount of N in the crop at harvest was insufficient to balance the N pools ((N uptake + Residual SMN) − (Initial N + N added)) and the difference was likely to have been a source for significant N loss via leaching.
- This information can be used to validate and improve modeling platforms (e.g., APSIM; https://www.apsim.info/) for future scenario testing to investigate a range of seasonal, soil type and management factors.

## Figures and Tables

**Table 1:** Feed quality components of catch crops at green-chop silage maturity at the Mossburn trial, Southland, in 2018. LSD represents the least significant difference at the 5% level within each column, *n* = 4. Means that share a letter in common are not statistically different.

Month of Sowing	Catch Crop Species	CP	SSS	Crude Fat	Lignin	NDF	ADF	Hemi-Cellulose	DOMD	ME	Total ME Harvested
		(%)	(%)	(%)	(%)	(%)	(%)	(%)	(%)	(MJ kg^−1^ DM)	(GJ ha^−1^)
June	Oats	11.8*d*	21.5*a*	2.25*b*	2.20*b*	51.8*b*	29.5*b*	22.3*c*	67.1*b*	10.73*b*	156.4*a*
	Ryecorn	13.7*cd*	12.7*d*	1.45*c*	3.75*a*	63.6*a*	35.7*a*	27.9*a*	53.3*d*	8.55*d*	67.8*c*
	Triticale	13.0*cd*	17.0*c*	1.43*c*	2.40*b*	60.8*a*	34.0*a*	26.7*a*	57.4*c*	9.20*c*	85.0*c*
August	Oats	12.7*cd*	22.3*a*	2.55*a*	2.03*b*	48.0*c*	27.5*c*	20.5*d*	72.1*a*	11.53*a*	155.2*a*
	Ryecorn	16.2*a*	17.1*c*	2.28*b*	2.03*b*	53.8*b*	29.5*b*	24.3*b*	66.7*b*	10.55*b*	108.2*b*
	Triticale	14.0*bc*	18.8*b*	2.25*b*	2.38*b*	51.7*b*	29.3*b*	22.4*c*	65.8*b*	10.53*b*	69.6*c*
LSD (5%)		2.2	2.4	0.22	0.49	3.1	1.8	1.6	2.7	0.44	17.3
*P* value		0.500	0.097	0.003	<0.001	0.021	0.01	0.079	0.002	0.003	<0.001
*Main effect means*											
Sowing date:											
June		12.8*a*	17.1*b*	1.71*b*	2.78*a*	58.7*a*	33.1*a*	25.7*a*	59.3*b*	9.49*b*	103.1*a*
August		14.3*b*	19.4*a*	2.36*a*	2.14*b*	51.2*b*	28.8*b*	22.4*b*	68.0*a*	10.88*a*	111.0*a*
LSD (5%)		1.3	1.4	0.13	0.28	1.8	1.0	0.9	1.5	0.25	10.0
*P* value		0.026	0.003	<0.001	<0.001	<0.001	<0.001	<0.001	<0.001	<0.001	0.112
Species:											
Oats		12.2*b*	21.9*a*	2.40*a*	2.11*b*	49.9*c*	28.5*c*	21.4*c*	69.6*a*	11.13*a*	155.8*a*
Ryecorn		15.0*a*	14.9*c*	1.86*b*	2.89*a*	58.7*a*	32.6*a*	26.1*a*	59.7*c*	9.56*b*	88.0*b*
Triticale		13.5*ab*	17.0*b*	1.84*b*	2.39*b*	56.2*b*	31.7*b*	24.6*b*	61.6*b*	9.86*b*	77.3*b*
LSD (5%)		1.6	1.7	0.16	0.35	2.2	1.3	1.1	1.9	0.31	12.2
*P* value		0.007	<0.001	<0.001	<0.001	<0.001	<0.001	<0.001	<0.001	<0.001	<0.001

CP = crude protein, SSS = soluble sugars and starch, NDF = neutral detergent fiber, ADF = acid detergent fiber, DOMD = disappearance of organic matter in dry matter, ME = metabolizable energy.

**Table 2:** Background soil fertility at planting (0–15 cm depth) at the catch crop trial site in Mossburn, Southland, in 2018.

Fertility Indicator	Average Site Value	Optimum Range ^1^
pH	5.7	5.7–6.2
Olsen P (mg L^−1^)	22	20–30
Exchangeable K (QT)	6	5–8
Exchangeable Ca (QT)	8	4–10
Exchangeable Mg (QT)	11	≥9
Exchangeable Na (QT)	2	10–12
CEC (me 100 g^−1^)	21	12–25

^1^ Based on values provided by RJ Hill Laboratories for cereal crops.

## Data Availability

Not applicable.
